# Supplementary material for: Gate-tunable superconducting diode effect in a three-terminal Josephson device
Source: Nat Commun. 2023 May 29;14:3078. doi: 10.1038/s41467-023-38856-0 (PMC10227039; doi:10.1038/s41467-023-38856-0)
Supplement: Supplementary file 1 — Supplementary Information [file 41467_2023_38856_MOESM1_ESM.pdf]

Supplementary Information for "Gate Tunable Superconducting Diode Effect in a Three-terminal Josephson Device"

Mohit Gupta,[1] Gino V. Graziano,[1] Mihir Pendharkar,[2,3] Jason T. Dong[4],
Connor P. Dempsey,[2] Chris Palmstrøm,[2,4,5] and Vlad S. Pribiag[1*]
[1]School of Physics and Astronomy, University of Minnesota, Minneapolis, Minnesota 55455, USA
[2]Electrical and Computer Engineering, University of California Santa Barbara, Santa Barbara, California 93106, USA
[3]Materials Science and Engineering, Stanford University, Stanford, California 94305, USA
[4] Materials Department, University of California Santa Barbara, Santa Barbara, California 93106, USA
[5] California NanoSystems Institute, University of California Santa Barbara, Santa Barbara, California 93106, USA
*To whom correspondence should be addressed; E-mail: vpribiag@umn.edu.

I. STABILITY OF RECTIFICATION

The value of critical current fluctuates over time as seen in Supplementary Fig.1**a**, this is believed to be due to fluctuation in the external magnetic field as the fluctuations are correlated for the positive and the negative direction. This results in the observed punch through errors. However, only 3 minor punch through errors , as seen in Supplementary Fig. 1 **b**, are observed over a time scale of 2hr, at an applied square wave of frequency 0.1Hz.

II. DATA FROM BIASING TERMINAL 2 AND 0

Here we show data when source terminal number is 2 and drain terminal is 0. In this case $I_1 = 0$ and $I_2 = I$ and $V_2 = V$. The Fraunhofer-like interference lobes are tilted in the opposite direction, as seen in Supplementary Fig. 2**a**, w.r.t. current axis compared to the case of terminal 1 being the source terminal as shown in Figure 3 **a**. For B just above just zero the $Q < 0$ as seen in supplymentry Fig. 2**b**, compared to the case of terminal 1 being source where $Q > 0$ for B just above 0. This is consistent with the expected behaviour from the network model.

III. RECTIFICATION IN DEVICE 2

Here we present data from a second device which is lithographically identical to Device 1. We measure this device with source terminal 2 and drain terminal 0, the observed Fraunhofer pattern (Supplementary Fig. 3**a**) is consistent with the device 1 at zero gate voltage. We can further access the two-terminal limit on this device by selectively gating the legs of the Y-shaped junction. By applying -7 V on $V_{\mathrm{g},1}$ and $V_{\mathrm{g},3}$ we pinch off the junction region between terminal pair 0-1 and terminal pair 1-2. While keeping the junction region between terminal pair 2-0 open by setting $V_{\mathrm{g},2}$ at 0 V. This effectively drives the device into a two-terminal regime as only one junction of the network is open. The observed Fraunhofer pattern is consistent with what has previously been observed for two-terminal Josephson junctions, as the lobes are not tilted (Supplementary Fig. 3**b**). The period in B also increases as the effective area of the junction has decreased. This is further clarified by evaluating δI_c for both the gate configurations as shown in Fig. 3**c**. Normalized δI_c (normalized by the critical current at zero applied field) is nearly zero in the two terminal regime as shown in red,

contrasted with the zero gate voltage case shown in blue.

We demonstrate of a square current wave (shown in yellow in Supplementary Fig. 3**d**) with an amplitude of $A = 0.2\ \mu$A, such that $I_c^- < A < |I_c^+|$. The device remains superconducting ($V \sim 0$ V) for the positive cycle of the square wave and has a finite voltage ($V \sim -8\ \mu$V) drop for the negative cycle. A single punch through error is observed over 5000 cycles. The critical current shows similar fluctuations in a similar way as seen for Device 1 (Supplementary Fig. 3**e**).

IV. MODEL FOR EVALUATING CRITICAL CURRENT

Transport in our Three-terminal device can be viewed more simply in terms of connected three two-terminal Josephson junctions of identical physical dimensions, as shown in Supplementary Fig. 4. Integration around contour C_1 gives the equation:

$$\frac{d\phi(x_1)}{dx_1} = \frac{2\pi B t}{\phi_0}$$

$$\phi(x_1) = \phi(x_1 = 0) + \frac{2\pi B t x_1}{\phi_0} \tag{1}$$

here t is the width of the junction, B is the applied magnetic field, and ϕ_0 is the flux quantum. Similarly for the other two Josephson junctions integration around C_2 and C_3 gives:

$$\phi(x_2) = \phi(x_2 = 0) + \frac{2\pi B t x_2}{\phi_0} \tag{2}$$

$$\phi(x_3) = \phi(x_3 = 0) + \frac{2\pi B t x_3}{\phi_0} \tag{3}$$

Integration around the contour C_e (shown in Fig. 4**a**) of area A gives the following equation:

$$\phi(x_1 = L) - \phi(x_2 = 0) - \phi(x_3 = 0) + \frac{2\pi}{\phi_0}(BA) = 0$$

$$\phi(x_1 = 0) = \phi(x_2 = 0) + \phi(x_3 = 0) - \frac{2\pi}{\phi_0}(BA + BtL) \tag{4}$$

If source terminal is S_1 and drain terminal S_0 we have from current conservation:

$$I_1 = \int_0^L J_c(x_1)\sin(\phi(x_1))dx_1 + \int_0^L J_c(x_3)\sin(\phi(x_3))dx_3 \tag{5}$$

$$\int_0^L J_c(x_2)\ \sin(\phi(x_2))dx_2 = \int_0^L J_c(x_3)\sin(\phi(x_3))dx_3 \tag{6}$$

Gate Voltage	t	S
$V_g = 0$ V	3.96 μm	5.17 μm^2
$V_g = -1.5$ V	4.5 μm	15.48 μm^2
$V_g = -2.5$ V	3.6 μm	-3.72 μm^2
$V_g = -4.1$ V	2.7 μm	0 μm^2

Supplementary Table I. Table of parameter values used to produce diode efficiency curves in Figure 4.

From Eq. 2,3,6:

$$\int_0^L J_c(x_2) \sin\left(\phi(x_2 = 0) + \frac{2\pi B t x_2}{\phi_0}\right) dx_2 = \int_0^L J_c(x_3) \sin\left(\phi(x_3 = 0) + \frac{2\pi B t x_3}{\phi_0}\right) dx_3 \quad (7)$$

To proceed in our calculations we assume $J_c(x_2) = J_c(x_3)$, then we must have $\phi(x_2 = 0) = \phi(x_3 = 0)$, this give Eq. 5 as:

$$\begin{aligned} I_1 = &\int_0^L J_c(x_1) \sin\left(2\phi(x_3 = 0) - \frac{2\pi}{\phi_0} BS + \frac{2\pi B t x_1}{\phi_0}\right) dx_1 \\ &+ \int_0^L J_c(x_3) \sin\left(\phi(x_3 = 0) + \frac{2\pi B t x_3}{\phi_0}\right) dx_3 \end{aligned} \quad (8)$$

The critical current can be evaluated by maximizing in $\phi(x_3 = 0)$ in Eq.8, the code used for maximization is provided with the manuscript. Due to flux focusing the width of the junctions t is much larger then the physical measured dimension of ~ 150 nm. The effective central area $S = (A + tl)$ is treated as an independent parameter in our simulations. The values of used t and S are listed in the Supplementary Table I and used $J_c(x)$ (modeled using Lorentzian distributions) are plotted in Supplementary Fig. 4 **c,d,e,f**. The code necessary for these calculations is provided with the manuscript.

V. TEMPERATURE DEPENDENCE OF DIODE EFFICIENCY

We also study temperature dependence of the diode effect by performing temperature sweep on Device 2 as shown in Supplementary Fig. 6. The device is measured in the positive polarity. The diode efficiency remains constant until the supercurrent itself starts

decreasing with temperature. This shows that the thermal fluctuations should not affect the performance of diode based upon multi-terminal Josephson junctions.

VI. DATA FROM DEVICE 4

Here we show data from Device 4 measured in a Bluefors dilution refrigerator at a base temperature of 8 mK. The junction dimensions are lithographically identical to Device 1 and 2, but no metal gates were deposited. The device is measured by biasing terminal 1 and 0, hence $I_1 = I$ and $I_2 = 0$ with $V_1 = V$. Obtained data is similar to what is obtained for device 1 (Supplementary Fig. 7 and Figure 3). This shows high degree of reproducibility and robustness of the diode effect in our three-terminal Josephson devices.

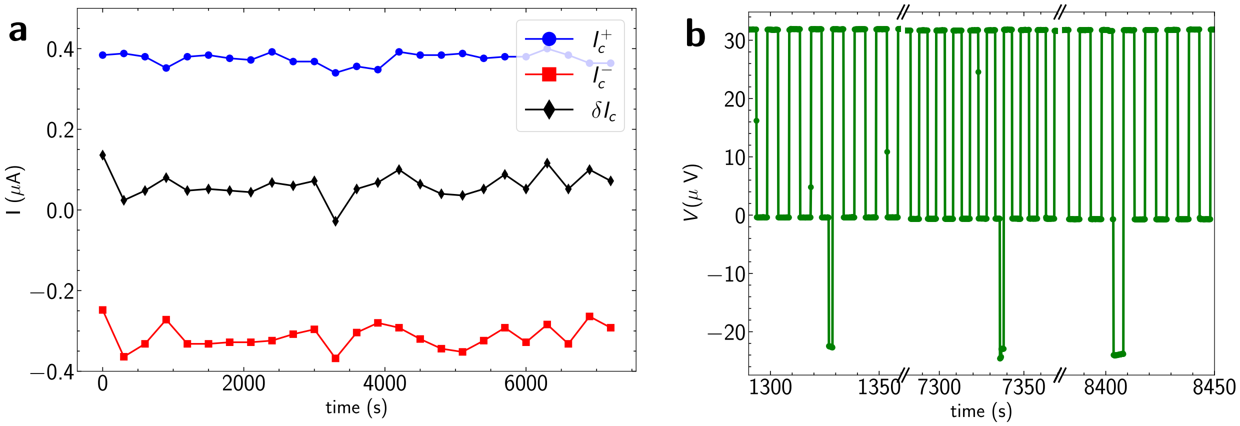

Supplementary Fig. 1. **Fluctuations in critical current: a** Critical current for both bias directions and δI_c as a function of time. **b** Voltage drop across the device for an applied square wave of frequency 0.1Hz, for the time intervals where punch through errors are observed.

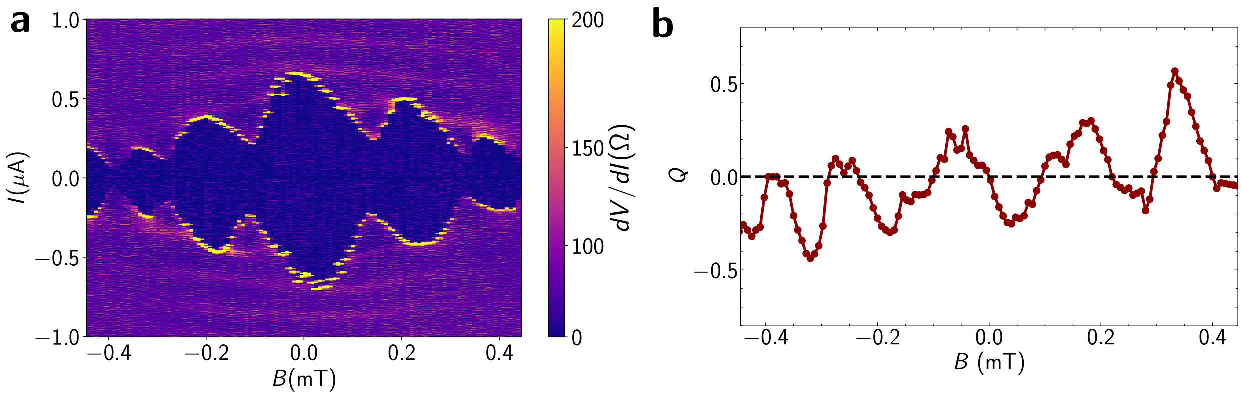

Supplementary Fig. 2. **Polarity flip by changing source drain configuration: a** Differential resistance map of device resistance as a function of bias current and applied out-of-plane magnetic field, B for source terminal 2 and drain terminal 0. **b** Q as a function of B extracted from panel **a**.

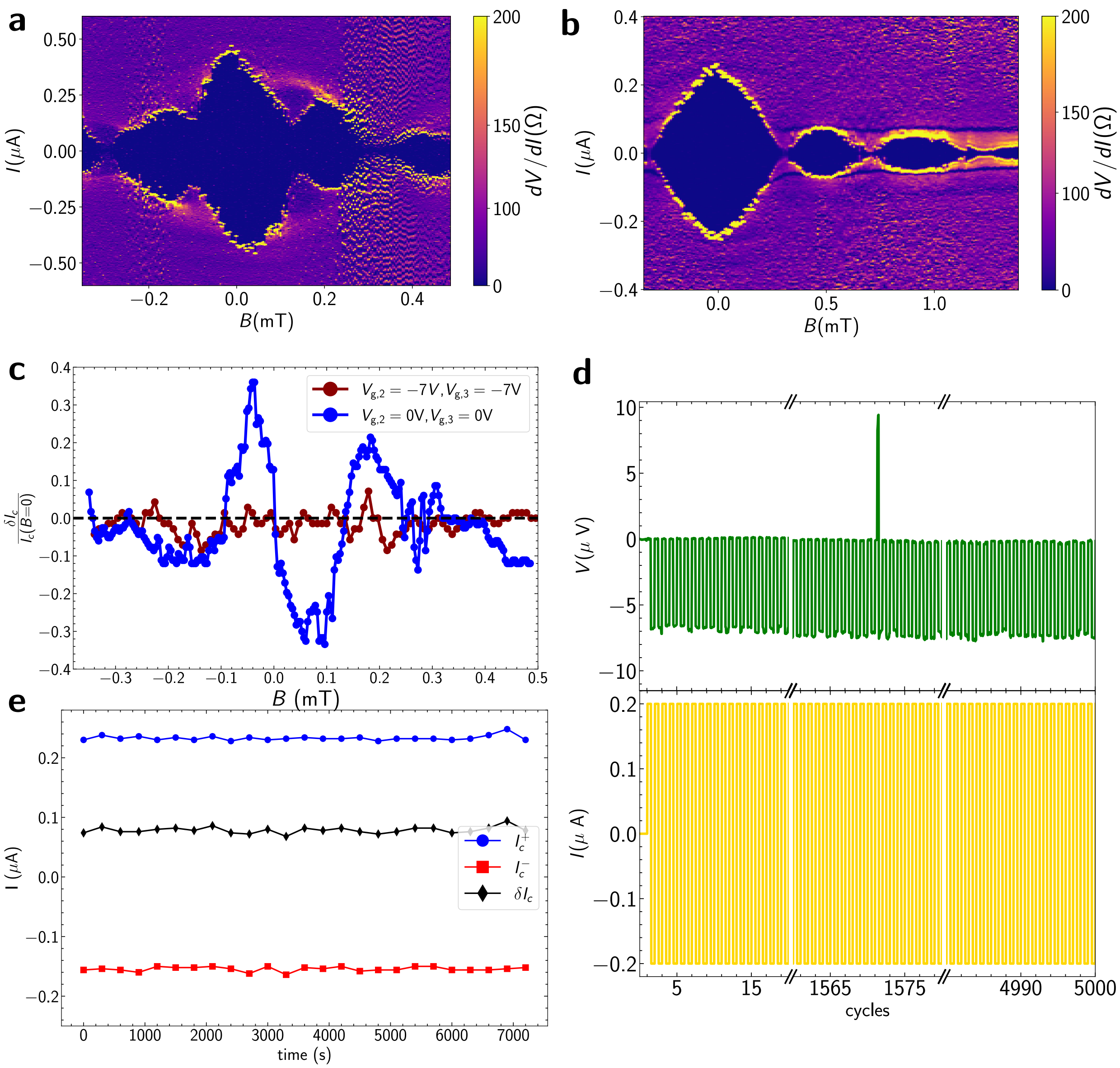

Supplementary Fig. 3. **Data from Device 2:** Differential resistance map of device resistance as a function of bias current and applied out-of-plane magnetic field, B for source terminal 2 and drain terminal 0 for **a** $V_{\mathrm{g},1} = 0\mathrm{V}, V_{\mathrm{g},2} = 0\mathrm{V}, V_{\mathrm{g},3} = 0\mathrm{V}$ and **b** $V_{\mathrm{g},2} = 0\mathrm{V}, V_{\mathrm{g},1} = -7\mathrm{V}, V_{\mathrm{g},3} = -7\mathrm{V}$. **c** δI_c extracted from panel **a,b** as a function of B. δI_c is normalized by $I_c(B = 0)$. **d** Voltage drop (shown in green) across the device for an applied square current wave (shown in yellow) for 5000 cycles over a measurement time of ∼15hr. **e** Critical current for both bias directions and δI_c as a function of time.

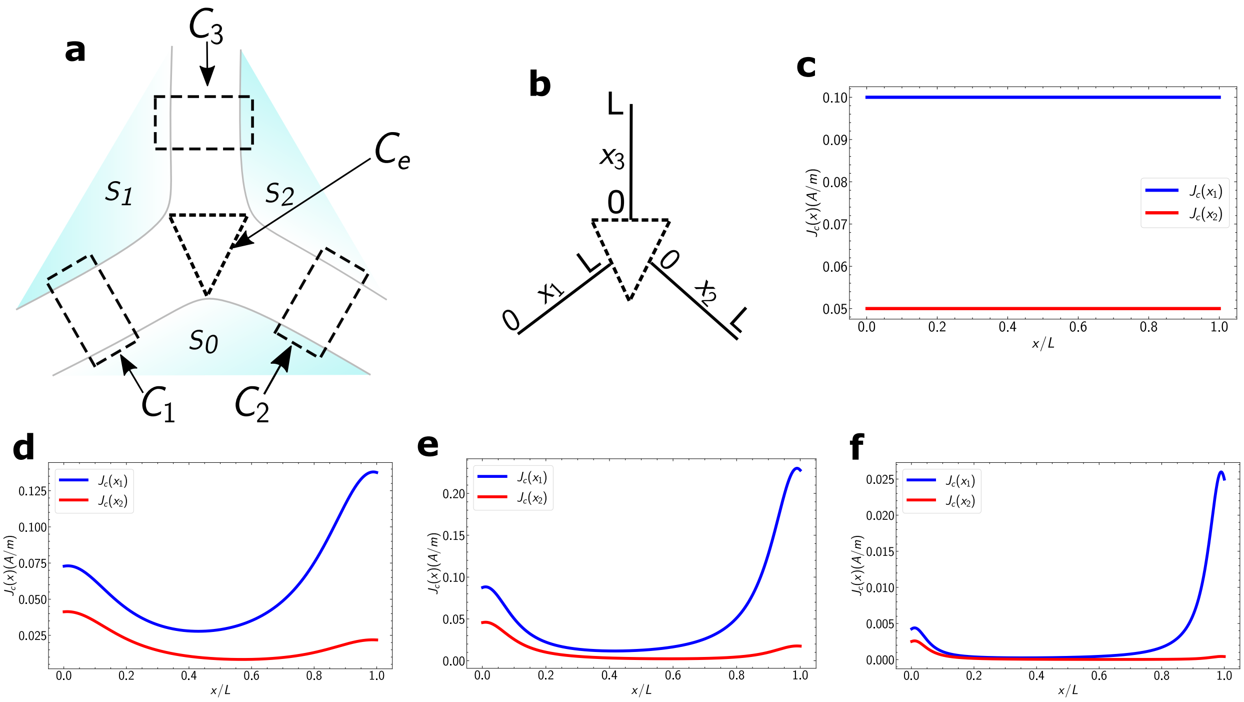

Supplementary Fig. 4. **Geometric Simulations:** **a** Schematic of the device, with integration paths shown in dashed lines for the model derivation. **b** Coordinate system used for the model derivation. supercurrent density profile of the junction for **c** $V_g = 0$ V **d** $V_g = -1.5$ V **e** $V_g = -2.5$ V **f** $V_g = -4.1$ V.

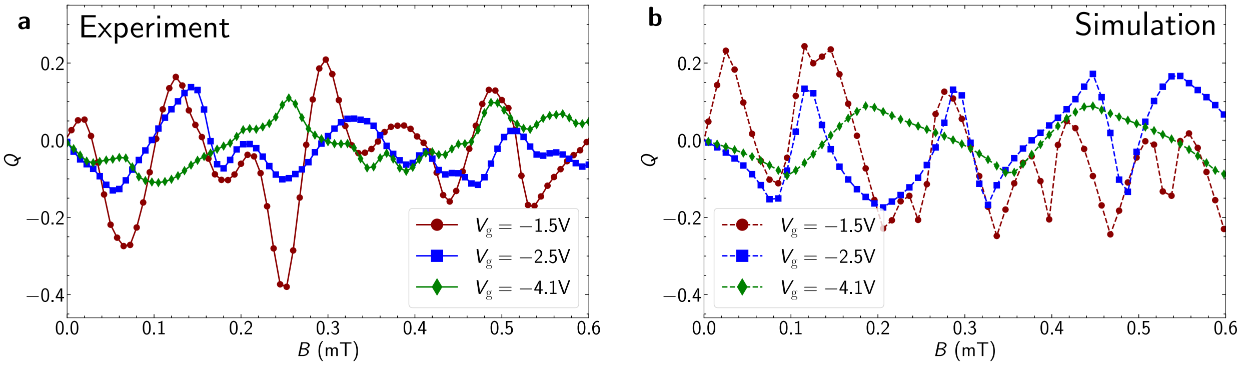

Supplementary Fig. 5. **Gate tunability of diode efficiency:** Diode efficiency factor Q as a function of B obtained **a** from experiment for different values of gate voltages and **b** from simulations.

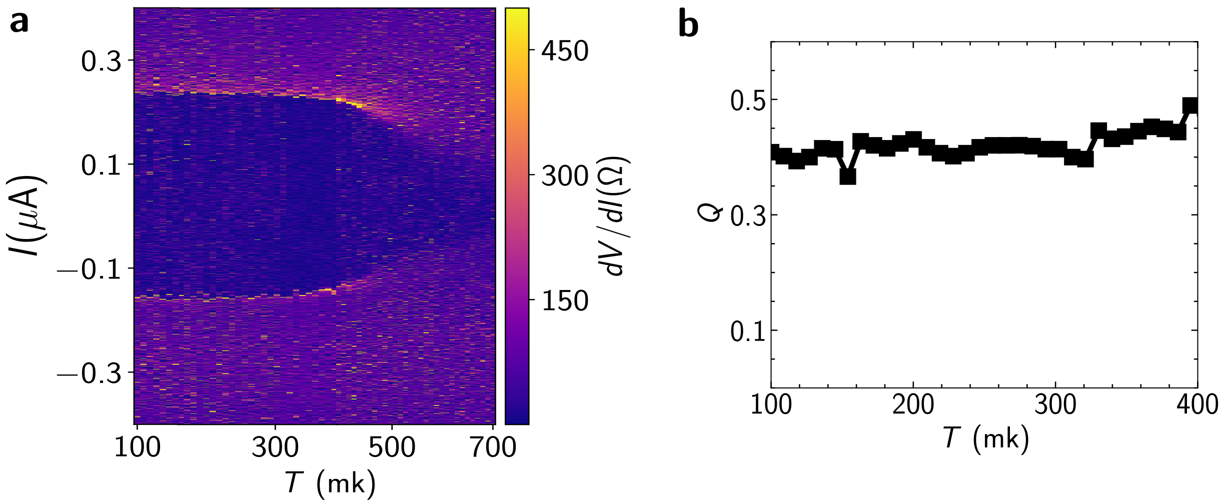

Supplementary Fig. 6. **Temperature dependence of diode effect: a** Differential resistance map of device resistance as a function of bias current and Temperature, T for source terminal 2 and drain terminal 0 in the positive polarity state. **b** Q as a function of T extracted from panel **a**.

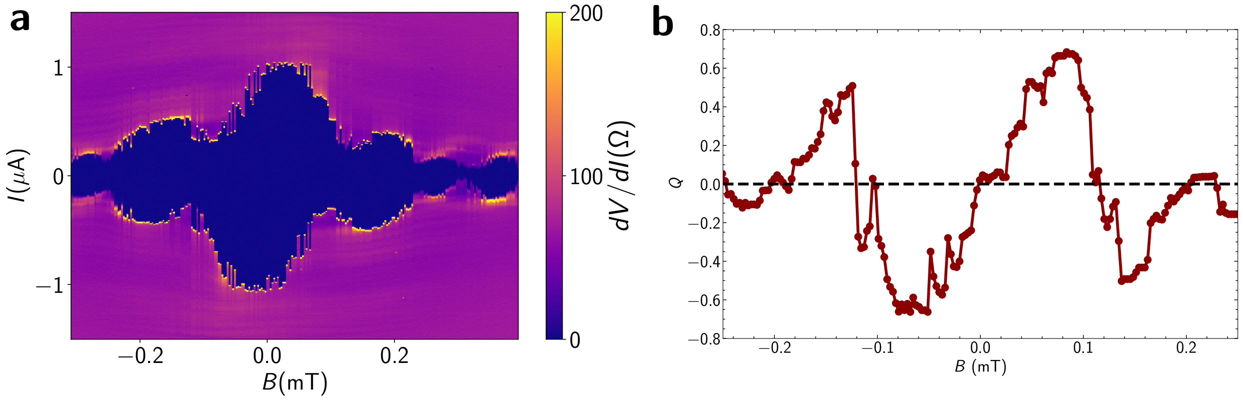

Supplementary Fig. 7. **Data from Device 4: a** Differential resistance map of device resistance as a function of bias current and applied out-of-plane magnetic field, B for source terminal 1 and drain terminal 0 for Device 3. **b** Q as a function of B extracted from panel **a**.
